# Strong Radiation Field Online Detection and Monitoring System with Camera

**DOI:** 10.3390/s22062279

**Published:** 2022-03-16

**Authors:** Yongchao Han, Shoulong Xu, Yang Liu, Ling Xu, Dawei Gong, Zhiwei Qin, Hanfeng Dong, Huaiqing Yang

**Affiliations:** 1 China Institute of Atomic Energy, Beijing 102413, China; xuling@ciae.ac.cn; 2 School of Resource Environment and Safety Engineering, University of South China, Hengyang 421001, China; qinzw@stu.usc.edu.cn (Z.Q.); hanfengdongusc@163.com (H.D.); usckane@163.com (H.Y.); 3 School of Mechanical and Electrical Engineering, University of Electronic Science and Technology of China, Chengdu 611731, China; ly2015@uestc.edu.cn (Y.L.); pzhzhx@uestc.edu.cn (D.G.)

**Keywords:** online radiation detection, monolithic active-pixel sensors, camera, strong radiation field, monitoring, CMOS

## Abstract

Herein, we report the γ-ray ionizing radiation response of a commercial monolithic active-pixel sensor (MAPS) camera under strong-dose-rate irradiation with an online detection and monitoring system for strong radiation conditions. We present the first results of the distribution of three types of MAPS camera and establish a linear relationship between the average response signal and radiation dose rate in the strong-dose-rate range. There is an obvious response signal in the video frames when the camera module parameters are set to automatic, but the linear response is very poor. However, the fixed image parameters are not good at adapting to the changes of the environment and affect the quality of the video frames. A dual module online radiation detection and monitoring probe was made to carry out effective video monitoring and radiation detection at the same time. The measurement results show that the dose rate detection error is less than 5% with a dose rate in the range of 60 to 425 Gy/h, and the visible light image does not have obvious distortion, deformation, or color shift due to the interference of the radiation response event and radiation damage. Hence, the system test results show that it can be used for online detection and monitoring in a strong radiation environment.

## 1. Introduction

The complex and strong radiation environment of a nuclear accident indicates stronger requirements for emergency detection and monitoring. How to quickly determine the radioactivity level at the accident site is an important prerequisite for radiation emergency response actions in the unknown and complex radiation field environment of a nuclear accident, such as nuclear facilities in the Fukushima accident. However, due to the complex working conditions of nuclear accidents, there is often radioactive pollution and strong ionizing radiation, which has a serious impact on emergency operations. For example, in Unit 2 of the Fukushima nuclear power plant accident, it was reported that the maximum radiation dose rate reached 530 Gy/h, which was estimated by analyzing the video images collected at the accident site. In recent years, research on radiation detection methods based on monitoring images and pixel sensors has become a hot topic in improving nuclear radiation detection technology. The application of monolithic active-pixel sensors (MAPS) to ionizing radiation detection is widely studied. In recent years, it has attracted much attention through application research on interventional radiology, the personal radiation dose monitoring of nurses and patients [1,2,3], charged particle track detection [4], and mobile phone radiation detection [5]. It shows great application value and potential in ultrawide range detection and strong radiation environment detection [6]. The key issue of MAPS radiation detection is to study its response characteristics to rays or particles. Experiments have confirmed that MAPS is sensitive to single ionized particles and generates radiation-responsive events in pixel arrays [7,8]. Different particles, such as X-rays, γ photons, and α and β particles, show its specific size and shape of typical response events in a frame [9]. The characteristic statistical parameters of response events are linearly related to the radiation dose rate [10,11]. Two-dimensional X and γ ray distribution imaging and the simultaneous measurement of the irradiation rate can be realized by using appropriate algorithms to process pixel values [12],^,^ as well as the radiation dose rate detection of low-energy gamma rays [13]. It has been reported that MAPS has good linear response characteristics for X-rays with energy greater than tens of keV. The dose rate detection uncertainty is less than 10% [1,2,3]. The detection accuracy depends on the calibration factor when there is no shielding structure and collimator, but the ray incidence angle has little effect on the detection results [5]. In terms of detection accuracy, for narrow beam photons, MAPS can obtain relatively consistent detection results with ionization chamber detectors [14]. Meanwhile, the MAPS, which adopts the pinned photodiode structure with four transistors, has strong radiation resistance [15], a wide detection range, and an upper limit of more than 1000 Gy/h [16]. However, this monitoring and detection system’s use in a strong radiation environment has not been reported, and there is no relatively mature commercial product on the market.

By analyzing the radiation effect of the cameras and the radiation response signal collected in the radiation environment, this paper designs an online detection and monitoring system for the strong radiation environment, based on the camera, and conducts radiation calibration and detection test experiments.

## 2. Experiments

### 2.1. Camera Samples

**Figure 1:**
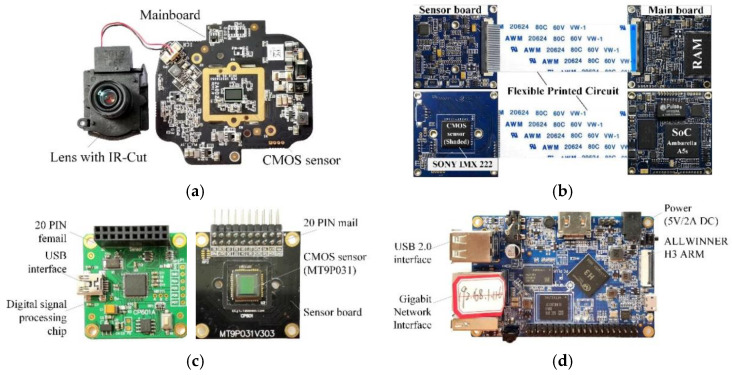
Camera module sample. (**a**) HIKVISION camera. (**b**) Sony IMX 222 module. (**c**) MT9P031 sensor board. (**d**) Orange Pi.

Three kinds of camera module were used for the radiation experiment. The DS-2CD1021FD-IW1 monitoring camera of HIKVISION, the IMX222 module of SONY, and MT9P031 module of APTINA were adopted. The physical diagram of the three types of modules is shown in Figure 1. The MT9P031 module included the sensor board, the digital signal processing board, and the Orange Pi system main board. The resolution of the MT9P031 sensor was 5 million. The sensor board only contained the CMOS image sensor and necessary passive components. The digital signal processing board was mainly used to collect image signals and perform certain preprocessing. The main board of the Orange Pi was mainly used for frontend processing and transmission of data.

During the experiment, all parameters of the HIKVISION camera were set to the default mode of the normal surveillance camera; that is, the parameters such as white balance, integration time, and gain were automatically adjusted. For the MT9P031 module and IMX 222 module, the white balance was fixed, the integration time and gain were set to manual adjustment, and all noise reduction functions were turned off. The setting parameters of all cameras during experiments were described in Table 1.

### 2.2. Experimental Setup

**Figure 2:**
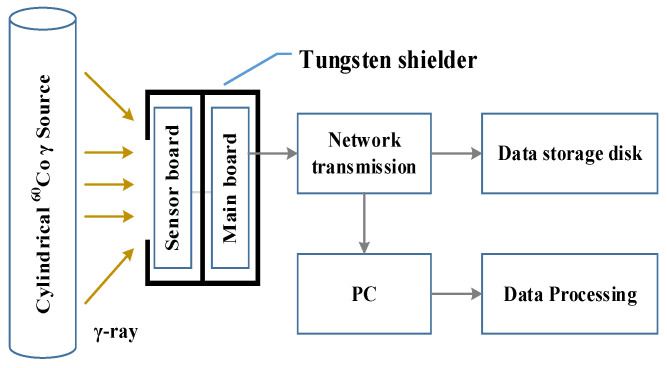
Experimental system diagram.

Radiation experiments were designed to study the radiation response of different cameras to gamma rays. The experimental system diagram is shown in Figure 2. A cylindrical ^60^Co γ-ray radiation source was used in the experiment with an energy of 1.17 MeV and 1.33 MeV and an activity of 3.33 × 10^14^ Bq. In order to prevent radiation damage of devices outside the photosensitive chip in the camera board, a tungsten shielding structure was used for radiation hardening. Only the sensors were exposed to γ-ray irradiation. All experiments were conducted at the China Institute of Atomic Energy (CIAE), all data were transmitted to the nonradiation area through the network transmission, and the data were stored and processed simultaneously.

Camera samples were placed above a slide rail, and the irradiated dose rate of the camera module was changed by adjusting the distance between the sample and the radiation source online. The total ionizing dose was measured using a radiochromic film dosimeter, and the dose rate was calculated as the ratio of the total ionizing dose to the irradiation time obtained; the measurement error was less than 5%. The experimental temperature was maintained at 21 °C. The data type was an 8-bit video at 25 fps, and the data were imported using MATLAB R2019a (Math Works Inc., Natick, MA, USA) and further split into individual frames. In the irradiation experiments of the HIKVISION camera and SONY IMX222 module, a video test card and LED light were used to test the camera’s acquisition of color images. Furthermore, during the MT9P031 module experiments, the dark images were captured by using a layer of opaque plastic material covered on front of the sensors to help insulate the sensor from contamination due to the surrounding visible light.

## 3. Results and Discussion

**Figure 3:**
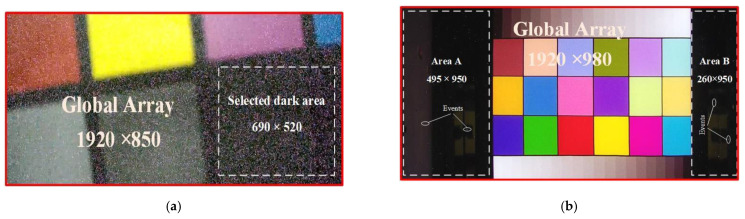
Color video frames during irradiation. (**a**) Color frame captured by HIKVISION camera. (**b**) Color frame captured by Sony IMX 222 module.

Figure 3 shows the color images collected by the SONY IMX 222 module at the dose rate of 77.3 Gy/h and HIKVISION camera at the dose rate of 83.0 Gy/h, respectively. The global array resolution of the Sony IMX222 module was 1920 × 980 and contained two dark areas. The resolution of the HIKVISION camera was 1920 × 850 contained only one dark area. It can be seen that the γ-rays formed white bright spots in the video frame, which were more obvious in the dark area. With the similar irradiation dose rate, the radiation response of the two types of cameras to γ-rays was significantly different. The HIKVISION module was more sensitive to γ-ray radiation, and the bright spots significantly affected the image quality.

**Figure 4:**
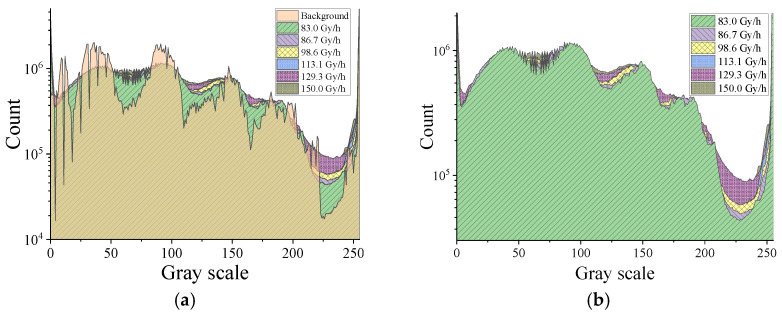
Histograms of the global array (**a**) and the dark area (**b**) of the frames captured by the HIKVISION camera.

Figure 4 shows the histograms of the global array (a) and dark area (b) of the HIKVISION camera under different irradiation conditions. As shown in Figure 4a, there were obvious differences in the global image histogram without and under irradiation, and the whole histogram during irradiation was smoother than that of the nonirradiated histogram. Both Figure 4a,b show the change to the histogram curve with different irradiation dose rates. The most sensitive ranges of the pixel value histogram were from 110 to 140, 160 to 180, and 200 to 250. For the selected dark area, the count of gray scale value from 200 to 250 showed an obvious difference with dose rate changes but still did not show a clear increase associated with a larger dose rate.

**Figure 5:**
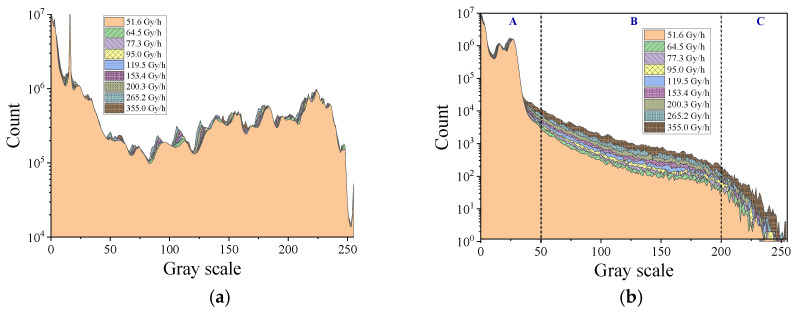
Histograms of the global array (**a**) and dark area (**b**) of the frames captured by the Sony IMX 222 camera with an integration time of 40 ms.

Figure 5 shows the histograms of the global array (a) and dark area (b) of the captured frames by the Sony IMX 222 camera with an integration time of 40 ms. It can be seen that both the histogram of the global array or the dark area show the characteristics of changing with the dose rate. Figure 5a mainly realized a peak increase and shift with a larger dose rate. For Figure 5b, the range of the histogram from 50 to 200 (area B) shows an obvious and regular increase with a larger dose rate, but it is unclear in the range of less than 50 (area A). Meanwhile, the curves are not smooth in the range greater than 200 (C area). We can conclude that the data of the gray scale value in the range from 50 to 200 reflects the radiation dose rate change the best.

**Figure 6:**
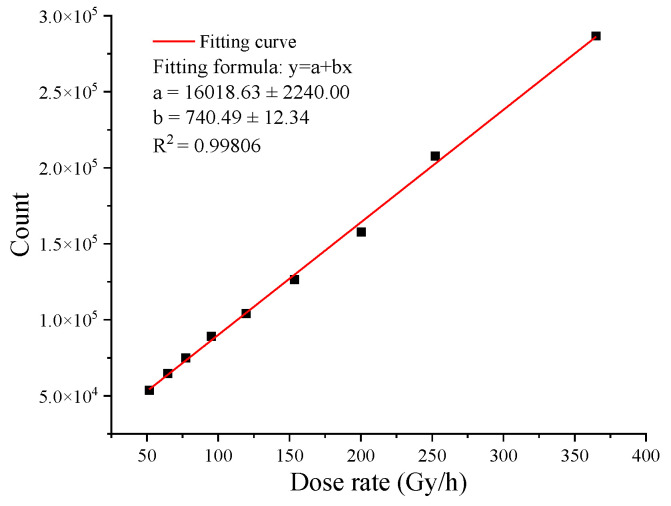
Radiation response result and fitting curve of the dark area of the frames captured by the Sony IMX 222 camera with an integration time of 40 ms.

As we can see from Figure 3b, a lot of the radiation response signal in the color image is drowned out by the visible signal. The radiation response events can only be observed in the dark background area of a color image. Therefore, when using color images for detection, it is necessary to extract and calculate the radiation response signal in the dark background area of the image. Figure 6 shows the radiation response result and fitting curve of the dark area (area A and area B) of the frames captured by the Sony IMX 222 camera with an integration time of 40 ms. We used average values from 50 to 200 to obtain this result. As shown in this figure, the fitting curve shows a significant linear response in the dose rate range from 50 Gy/h to 400 Gy/h; the linearity of the fitting curve is R^2^ = 0.99806.

**Figure 7:**
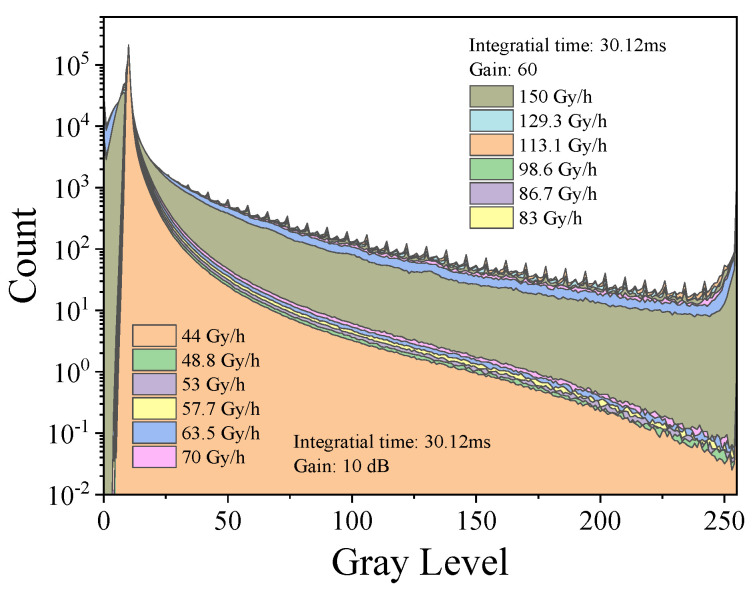
Histograms of dark images of the frames captured by the MT9P031 module.

The histograms of the dark images of the frames captured by the MT9P031 module are shown in Figure 7. As we can see, there was an obvious hierarchical increase in the histogram with a larger irradiation dose rate. This increase was also clear in the range from 50 to 200. Meanwhile, under the condition of the same integration time, the shape of the histogram changed significantly with the gain exchange. With a large gain, the count of pixels with a gray value of less than 20 decreased, but the count of pixels with a gray value greater than 20 increased. At the same time, the histogram curve in the range of 50 to 200 was smoother with a larger increase. This may have been due to the fact that the weak response events were amplified when the gain was increased, and all the pixel values increased significantly. However, because of this, more pixels were approaching saturation, which can explain why the count of the pixels with a value near to 255 increased with a larger gain.

**Figure 8:**
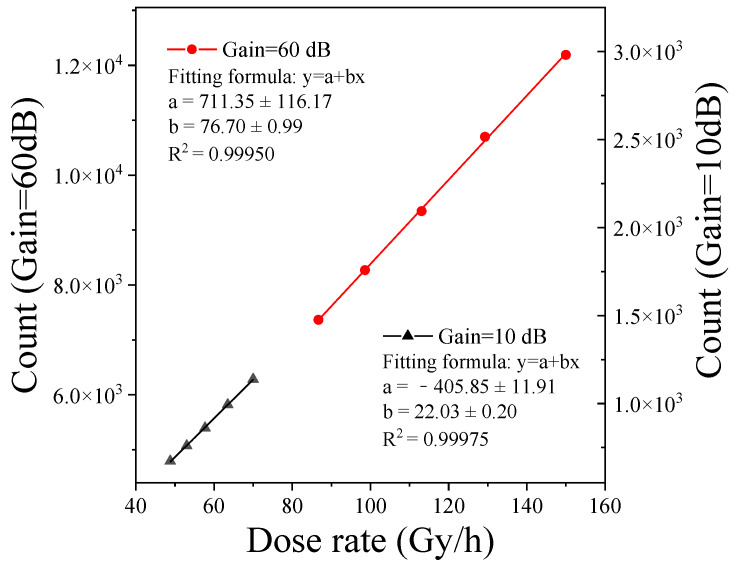
Radiation response curve of the dark frames captured by the MT9P031 module.

Therefore, we can obtain the response curve of the MT9P031 module under different gains by counting the data in Figure 7. Figure 8 shows the statistical results and fitting curve of the MT9P031 module with the gain as 10 dB and 60 dB. Both fitting curves reflect great linear response characteristics under various gain conditions, and the linearity R2 is greater than 0.999.

## 4. System Setup and Test

### 4.1. Detection and Monitoring System Setup

**Figure 9:**
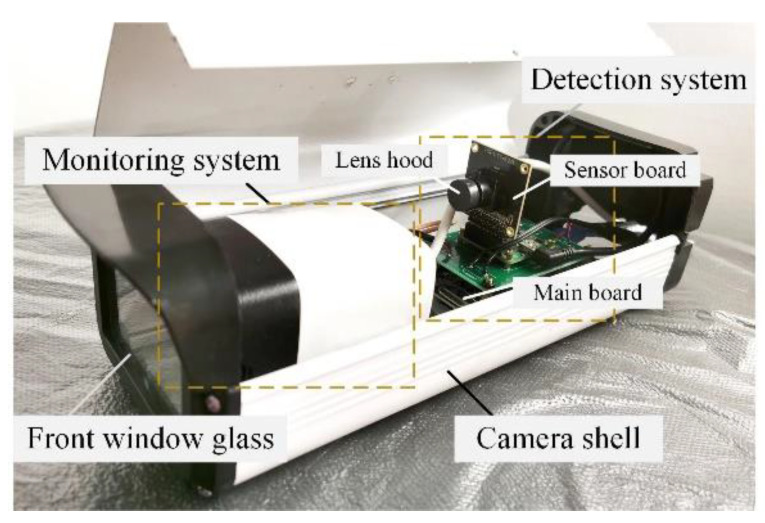
Physical drawing of the detector system probe.

**Figure 10:**
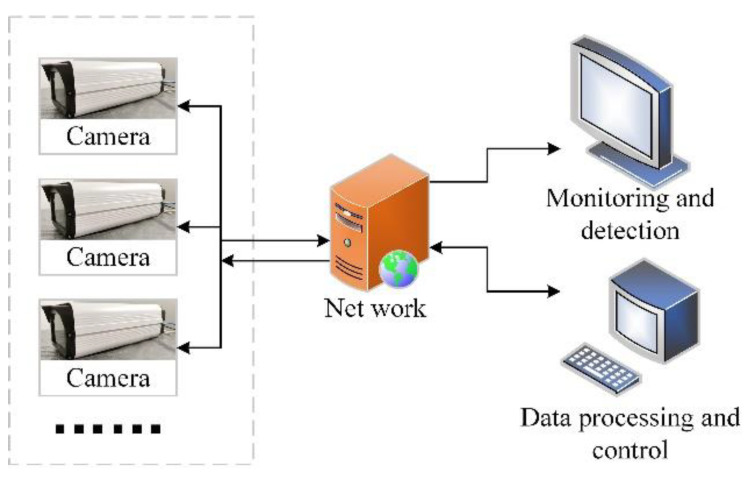
Diagram of the online radiation detection and monitoring system.

In order to carry out effective video monitoring and radiation detection at the same time, a dual-module online radiation detection and monitoring probe was made. The structure of the probe is shown in Figure 9. The overall shell structure used a box camera structure. The monitoring system was placed in the front of the probe, and the detection system was placed in the rear. The front glass and the box shell were used to fix the system structure and protect the devices. The monitoring system adopted a SONY IMX 222 camera, and the detection system included a MT9P031 sensor module and an Orange Pi system main board. The data were output from the monitoring camera’s main board and the Orange Pi board, respectively, by the network cable and transmitted to the network server. Multiple detectors were formed into a monitoring and detection network; both the video data and detection data were processed by the network server and displayed on the client side. The diagram of the online radiation detection and monitoring system is shown in Figure 10.

### 4.2. System Test

**Figure 11:**
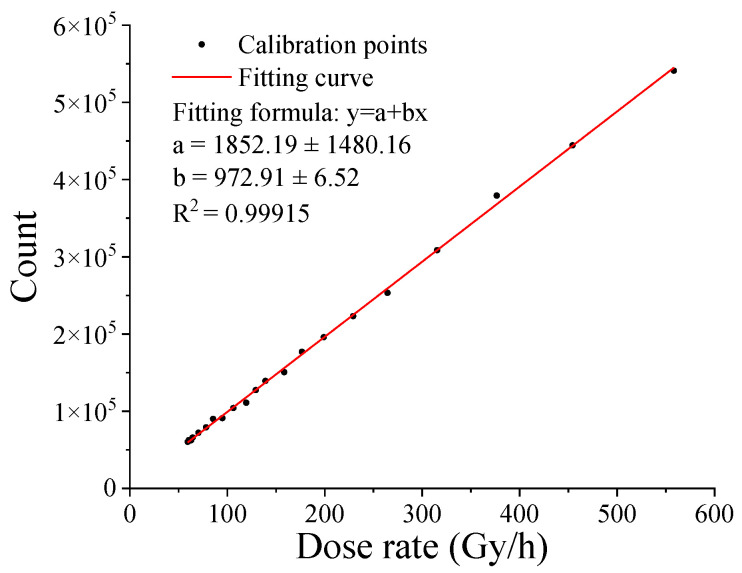
Radiation detection scale curve of the system.

**Figure 12:**
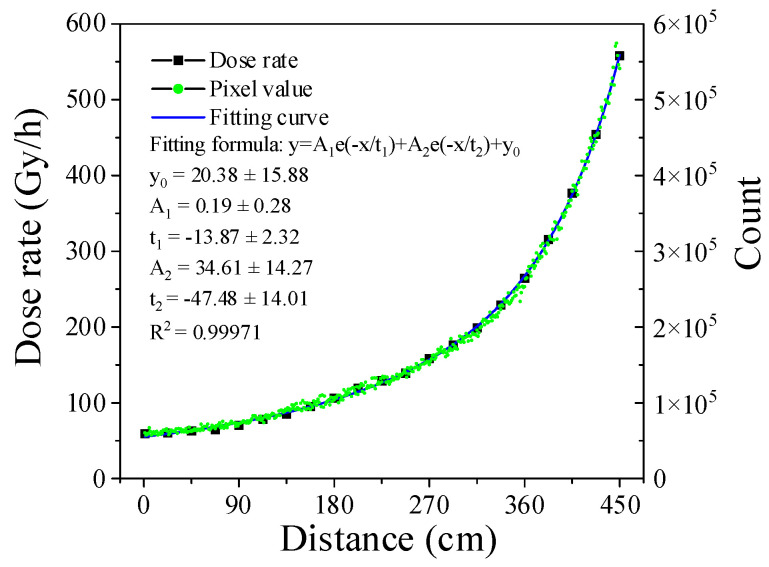
Continuous scale and calibration curve.

During the system operation, the video data were stored and transmitted in the form of video images, the detection data were preprocessed by the Orange Pi system, which only output the histogram data of the frames. The data in the range of 50 to 200 were selected for statistical calculation. The system was online-calibrated with a slide rail. Figure 11 shows the calibration data of the dose rate in the range of 50 to 600 Gy/h, and the linear fitting results are also shown in this figure. To verify the calibration data, as shown in Figure 12, a method of continuous motion data collection was used for verification. During verification, the sampling rate was 25 frames per second, and every two frames were counted once. The verification results showed that the statistical results of the two frames of data fully met the detection requirements, and the response curve fitted well, as shown in the figure.

After the system was calibrated, a radiation experiment was carried out. The test results are shown in Table 2. The respective measurement results and detection results were compared. The test experiment results showed that, after comparing the results between the system detection and dosimeter measurement, the dose rate detection error was less than 5%. The system test results showed that it can be used for online detection and monitoring in a strong radiation environment.

## 5. Conclusions

In this paper, it was explained that a commercial monolithic active-pixel sensor (MAPS) camera is sensitive to γ-ray ionizing radiation and can be utilized for direct detection. The linear response was poor when the camera module parameters were set to automatic, even though there was still an obvious response signal in the video frames. There was a significant linear response characteristic when the parameter settings of the color image were fixed. However, the fixed image parameters were not good at adapting to the changes in the environment and affected the quality of the video frames. This issue was solved by making a dual-module online radiation detection and monitoring system. The MT9P031 module was used for dark-image radiation measurement with a representative range of 50 to 200 in the histogram. In addition, the SONY IMX 222 camera was adapted to video monitoring. Hence, the system can be used to accurately measure the radiation dose rate in real time and, thus, can be used as a monitoring and detection sensor for robot systems in strong-radiation field conditions.

## Figures and Tables

**Table 1:** The setting parameters of cameras during experiments.

No.	Camera	Type of Image	Parameter Set			
			White Balance	Integration Time	Gain	Noise Reduction
1	MT9P031 module	Color image	Fixed	0.4 ms	6 dB	Shut off
2	HIKVISION camera	Color image	Auto	Auto	Auto	Auto
3	SONY IMX222 module	Dark image	Fixed	0.4 ms	6 dB	Shut off

**Table 2:** Test results of the online radiation detection and monitoring system.

No.	Dosimeter Measurement Result (Gy/h)	System Detection Result (Gy/h)	Error (%)	Monitoring Image	No.	Dosimeter Measurement Result (Gy/h)	System Detection Result (Gy/h)	Error (%)	Monitoring Image
1	60.6	59.38	2.01	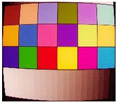	4	218.05	221.83	1.73	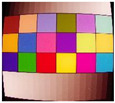
2	89.91	90.97	1.18	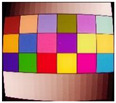	5	340.32	334.20	1.80	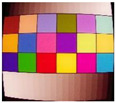
3	132.61	127.95	3.51	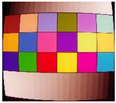	6	421.25	418.92	0.55	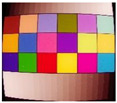

## Data Availability

Not applicable.
